# Influence of slag composition on the stability of steel in alkali-activated cementitious materials

**DOI:** 10.1007/s10853-017-1919-3

**Published:** 2017-12-18

**Authors:** Maria Criado, Susan A. Bernal, Pablo Garcia-Triñanes, John L. Provis

**Affiliations:** 1 Department of Materials Science and Engineering, The University of Sheffield, Sir Robert Hadfield Building, Sheffield, S1 3JD UK; 2 Wolfson Centre for Solids Handling Technology, University of Greenwich at Medway, Chatham Maritime, ME4 4TB UK

## Abstract

Among the minor elements found in metallurgical slags, sulfur and manganese can potentially influence the corrosion process of steel embedded in alkali-activated slag cements, as both are redox-sensitive. Particularly, it is possible that these could significantly influence the corrosion process of the steel. Two types of alkali-activated slag mortars were prepared in this study: 100% blast furnace slag and a modified slag blend (90% blast furnace slag + 10% silicomanganese slag), both activated with sodium silicate. These mortars were designed with the aim of determining the influence of varying the redox potential on the stability of steel passivation under exposure to alkaline and alkaline chloride-rich solutions. Both types of mortars presented highly negative corrosion potentials and high current density values in the presence of chloride. The steel bars extracted from mortar samples after exposure do not show evident pits or corrosion product layers, indicating that the presence of sulfides reduces the redox potential of the pore solution of slag mortars, but enables the steel to remain in an apparently passive state. The presence of a high amount of MnO in the slag does not significantly affect the corrosion process of steel under the conditions tested. Mass transport through the mortar to the metal is impeded with increasing exposure time; this is associated with refinement of the pore network as the slag continued to react while the samples were immersed.

## Introduction

The growth in emphasis on reduction of environmental impact has led the construction sector to become very interested in the development of new alternative cements. One promising approach is based on aluminosilicate raw materials such as blast furnace slag derived from the iron making process, which can be reacted with an alkali source to produce binding materials known as alkali-activated cements [1, 2].

Corrosion of reinforcing steel is the main cause of premature degradation of reinforced concrete structures. The passivity of reinforcing steel in concrete, whether based on Portland cement (PC) or alkali-activated cements, is attributed to the formation of a thin passive film on the steel surface. This film is maintained by the high pH of the surrounding concrete, unless the film is damaged by the presence of chloride ions or by a pH drop due to carbonation of the concrete [3, 4]. Carbon steel reinforcement has been reported to be compatible with alkali-activated mortars, where corrosion rates can be similar to or lower than those recorded in PC mortars [5, 6]. However, it is essential to understand the influence on steel corrosion of the redox-active elements supplied by the metallurgical slags that are used as precursors to alkali-activated cements, as these elements may interact with the passive film and change the rate and/or mechanism of steel corrosion in the presence of chloride.

Among the minor elements found in the slags that have been used to produce alkali-activated cements, sulfur and manganese must be viewed carefully regarding electrochemical interactions, as both are redox-sensitive elements. Silicomanganese (SiMn) slag is a by-product of the production of silicomanganese alloys by carbothermal reduction of manganese in a submerged electric arc furnace. SiMn slag is characterized by a relatively high content of manganese oxide (~ 10 wt%), and it is usually rich in SiO_2_ (38–43 wt%), CaO (25–30 wt%) and Al_2_O_3_ (12–15 wt%), with lower levels of MgO (2–5 wt%) [7–9]. Herasymenko [10] observed that manganese was distributed throughout the bulk SiMn slag matrix, although there were small zones enriched in Mn. This author also detected S throughout the glass, and the sulfides did not seem to have affinity for Mn.

Manganese, in nature, is often found in close association with iron. Fe and Mn may transform between solid and dissolved forms depending on the redox conditions and are oxidized by atmospheric oxygen, iron to a valence of + 3 and manganese to a valence of + 4, forming insoluble oxides and hydroxides. The divalent ions of both elements may also precipitate as hydroxides, carbonates, silicates and sulfides; such iron compounds are more insoluble than those of manganese, especially for sulfides [11]. The reducing environment generated through alkali activation of SiMn slag is expected to be comparable to that formed when blast furnace slag is dissolved in an alkaline medium, due to the release of sulfides.

During hydration of sulfide-containing cements, such as those containing blast furnace slag, the sulfide can be partially oxidized to sulfate during the hardening process [12], although it has also been noted that the characteristic blue-green color of many alkali-activated slag cements is related to the presence of polysulfide radical anions [13]. Standard reduction potentials show that Mn^3+^ is the most powerful oxidant in this cementitious system (SO_4_^2−^/S^2−^ 0.33 V, O_2_/2OH^−^ 0.40 V, Fe^3+^/Fe^2+^ 0.77 V and Mn^3+^/Mn^2+^ 1.51 V) and so, together with Fe^3+^, will be reduced to Mn^2+^ and Fe^2+^, respectively [12].

Therefore, it is important to understand the corrosion process of steel rebars embedded in concretes containing SiMn slag, where incompletely oxidized Mn species can act as an oxidant, modifying the chemistry of the passivation and corrosion processes taking place at the steel–cement interface. The aim of this work is to study the corrosion of steel in alkali-activated blast furnace slag and in a modified alkali activated mortar system (blast furnace slag + 10% SiMn slag), to determine the influence of the variation of the redox potential on the stability of steel passivation under exposure to alkaline and alkaline chloride-rich solutions, through electrochemical testing.

## Experimental methodology

### Raw materials

A commercial powdered blast furnace slag (BFS) supplied by ECOCEM^®^, France, and a silicomanganese slag supplied by Ferroatlantica S.L. (Spain) were used; their oxide compositions as determined by X-ray fluorescence (XRF) are shown in Table 1. The silicomanganese slag had a significantly lower calcium content than BFS, but very high silica and manganese contents.

**Table 1:** Chemical composition of the raw materials used, obtained by XRF

Oxides^a^ (wt%)	Blast furnace slag	Silicomanganese slag
CaO	41.8	25.6
SiO_2_	36.0	42.8
Al_2_O_3_	11.3	13.1
MgO	6.5	3.7
SO_3_	0.7	0.7
Fe_2_O_3_	0.3	0.1
TiO_2_	0.5	–
MnO or Mn_3_O_4_	0.3 as MnO	10.6 as Mn_3_O_4_
K_2_O	0.4	1.2
Others	0.3	1.9
LOI	1.95	0.14

LOI is the loss on ignition at 1000 °C

^a^S, Fe and Mn are actually likely to be largely reduced in the slags, but they are represented as oxides in the XRF analysis. For the blast furnace slag, chemical analysis according to EN 196-2 showed that 49% of the sulfur was present as sulfate and the remainder as sulfide

Silicomanganese slag was ground using a Pukka ring mill pulverizer to reduce the as-received coarse granules to a fine powder. The average particle size was 11.2 ± 0.1 µm and 77.8 ± 0.4 µm for blast furnace slag and silicomanganese slag, respectively (average values of four measurements), determined by laser diffraction using a dry dispersion unit. The Blaine finenesses determined for blast furnace slag and silicomanganese slag were 506 ± 22 m^2^/kg and 295 ± 10 m^2^/kg, respectively (average values of four measurements) [14]. This test is based on the relationship between the rate at which air can pass through a packed bed of particles under a given pressure drop, at the powder particle size (and hence surface area). The surface area is determined by calibration of the instrument using powders of a known surface area and particle shape. The activator was prepared by pre-dissolving commercial sodium metasilicate powder (Sigma-Aldrich, Na_2_SiO_3_) into distilled water until complete dissolution was reached.

Mild steel rebars (diameter 12 mm) according to BS 4449:2005 + A3:2016 [15] were used for the electrochemical tests. Their chemical composition (% by weight) was 0.18–0.22 C, 0.23 Si, 0.76 Mn, 0.04 P, 0.03 S, 0.13 Cr, 0.20 Ni, 0.47 Cu, 0.02 Mo and balance Fe. The ribbed rebars were cut to produce 100 mm-long sections for testing. These were embedded in mortars with the surface in as-received condition, with the ends masked with Sikagard-62 (Sika, UK) epoxy resin coating to leave an exposed surface area of 10 cm^2^ per bar.

### Mortar sample preparation

To produce the mortars, both 100% blast furnace slag (BFS) and a mixture of 90% blast furnace slag and 10% silicomanganese slag were activated with a 7 wt% dose of sodium metasilicate (i.e., 7 g of Na_2_SiO_3_ per 100 g of slag), at a liquid to solids (anhydrous slag + anhydrous sodium metasilicate) ratio of 0.40. A CEN standard sand (EN 196-1 [16]) was used, and the sand/slag ratio was held at 3.0. Samples were cast in steel molds to the dimensions described in the “Methods” section for each technique applied, kept sealed in the molds for 24 h at 20 ± 2 °C, then demolded, sealed with cling film and cured at 20 ± 2 °C for the specified time period, before they were subjected to different tests.

### Methods

#### Compressive strength and mercury intrusion porosimetry (MIP)

The compressive strength of mortar specimens was determined according to an adapted form of ASTM C109/C109M-16a [17] (adapted to provide a mixing protocol suitable for alkali-activated specimens) with a loading rate of 0.25 MPa/s, using triplicate 50 mm cube samples.

Mercury intrusion porosimetry (MIP) was used to provide information regarding the pore size distribution and pore volume of the mortars. Several fragments were obtained from the middle of mortar specimens, immersed in isopropanol for 14 days, then placed into a desiccator and evacuated for 2 days [18]. The pore diameter was derived using Washburn’s law: $D = \left(-4\,{\text{cos}}\,{\theta}\right){\gamma}/P$, where *D* is the pore diameter (µm), *θ* the contact angle between the fluid and the pore mouth (130°; [19]), *γ* the surface tension of the fluid (485 mN/m; [19]), and *P* the applied pressure to fill up the pore with mercury (MPa). The porosimeter employed was a Micromeritics Autopore V 9600, with a maximum pressure of 208 MPa.

#### Chloride migration

The non-steady-state chloride migration was conducted using Nordtest NT Build 492 [20]. Duplicate disk samples, 100 mm diameter × 50 mm height, were tested after 28, 90 and 180 days of curing, with AgNO_3_ colorimetric analysis applied at the end of the test and results analyzed according to the equation specified in the test protocol. Chloride migration depth is measured as the visible boundary between white precipitation of AgCl when chloride is present in sufficient quantities that this is the dominant reaction product, and precipitation of brown Ag_2_O otherwise [21].

#### Electrochemical testing

The assessment of the corrosion resistance of steel in alkali-activated BFS and BFS-SiMn cements was carried out using prismatic mortar specimens (80 × 50 × 50 mm) with two embedded mild steel rebars (12 mm diameter and 100 mm length). Four mortars were prepared for each condition because the (destructive) determination of polarization curves was carried out after 60 and 150 days of immersion and two mortars were necessary for each age; the measurements were taken in duplicate to give four total rebars for each condition. After reaching 28 days of curing, the cling film was removed and the prismatic samples were exposed to the following conditions

- Samples were kept sealed in the standard laboratory conditions (21 ± 3 °C; 50–70% relative humidity) (denoted SL).
- Immersed in 1 M NaOH, as a reference condition resembling the alkalinity of the pore solution of alkali-activated slag cements [22] (denoted N).
- Immersed in alkaline chloride solution: samples immersed in 1 M NaOH + 3.5 wt% NaCl (denoted Cl)

The pH values of the immersion media were monitored at least once every 30 days and remained invariant (pH ~ 14) during the duration of the test for conditions N and Cl.

**Figure 1:**
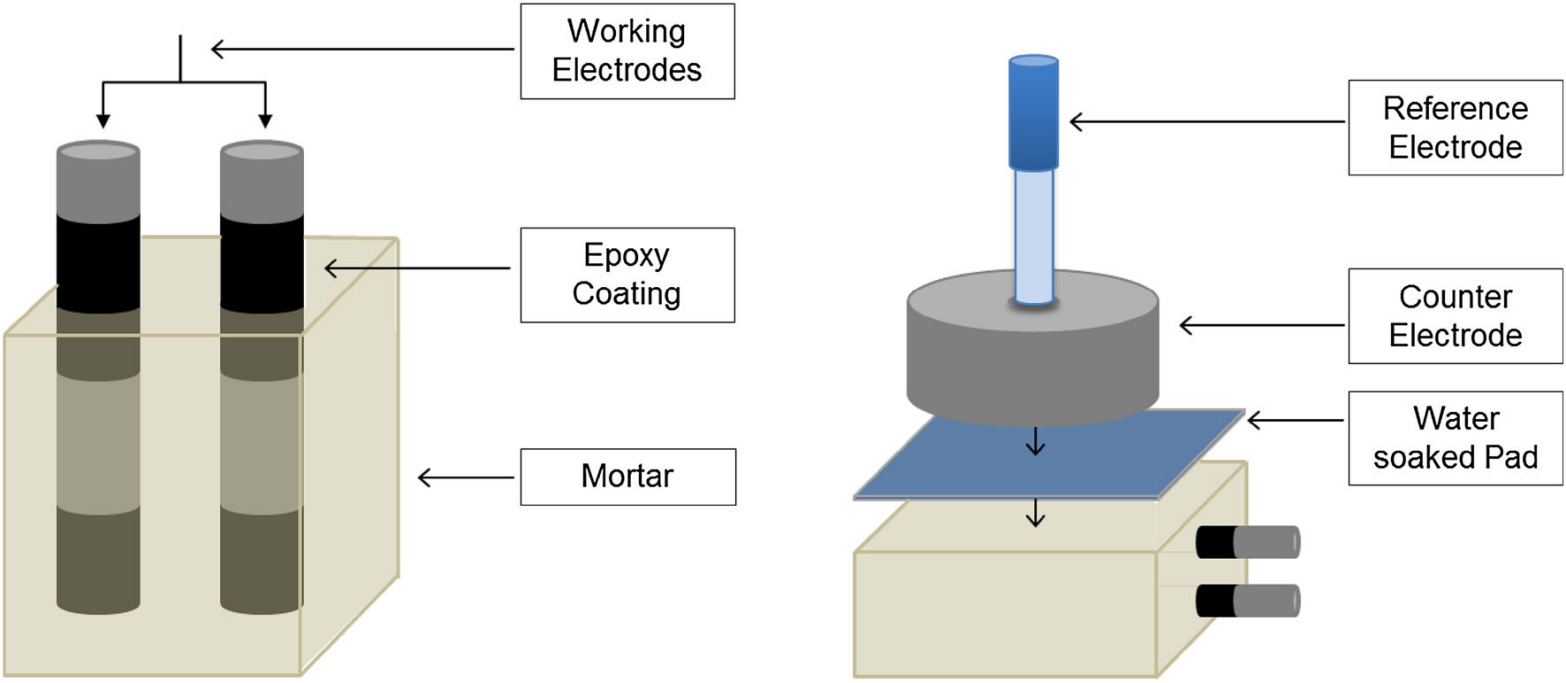
Reinforced prismatic sample and electrochemical testing setup

Corrosion potential (*E*_corr_) and electrochemical impedance spectroscopy (EIS) data were recorded periodically up to 150 days, and polarization curves were measured after 60 and 150 days of immersion in each environment. A conventional three-electrode cell was used for electrochemical measurements. The steel bars embedded in the prismatic mortar specimens acted as working electrodes, and a stainless steel cylinder was placed above the mortar to act as the counter electrode. The counter electrode used had the same size as the mortar, achieving a uniform distribution of the current lines and avoiding the issues of field non-uniformity that are sometimes induced when a counter electrode is placed on top of the specimen [6, 23]. The counter electrode had a centrally drilled hole, where an Ag/AgCl (filled with 3 M KCl) electrode was positioned to act as the reference electrode. A pad soaked in tap water was used to facilitate the electrical measurements. Figure 1 shows the electrochemical testing setup.

A Princeton Applied Research VersaSTAT 3F was used to conduct the electrochemical measurements. EIS measurements were recorded at *E*_corr_ in a frequency range from 1 MHz to 1.58 mHz with a logarithmic sweep of 5 points per decade. EIS involved imposing a 10-mV rms amplitude excitation voltage. After 60 and 150 days of immersion, ohmic drop-compensated anodic polarization curves were recorded, at a scan rate of 0.1667 mV s^−1^. EIS and polarization curve measurements were taken after the *E*_corr_ was stabilized for at least 30 min.

#### Corrosion product analysis

After 60 days and 150 days of immersion, rebars not used for anodic polarization curve determination were extracted from the mortars for visual observation and Raman spectroscopic analysis of surface corrosion products. The slabs were carefully broken with a chisel and the steel bars extracted. Raman measurements were taken using a Renishaw InVia Raman Microscope equipped with a 514.5-nm laser, a Leica microscope, and 50 × magnification objective lenses. The laser power was 1 mW, the integration time was 15 s, and 3 accumulations were used. The Raman shift was calibrated before measurements according to the silicon peak at 520 cm^−1^.

## Results and discussion

### Mortar characterization

The compressive strengths of alkali-activated BFS and SiMn mortars after 7 and 28 days of curing are reported in Table 2. The 7-day compressive strength values were similar for both mortars, but BFS mortars showed slightly higher compressive strength values at later ages; the SiMn slag used here is both intrinsically less reactive [24] and has a larger particle size, than the BFS used.

**Table 2:** Compressive strength and chloride migration coefficients of alkali-activated BFS and SiMn mortars

Sample ID	Curing days	Compressive strength (MPa)	Chloride migration coefficient, *D*_nssm_ (× 10^−12^ m^2^/s)
BFS	7	55.1 ± 1.3	–
	28	79.0 ± 1.2	1.45 ± 0.38
	90	81.8 ± 2.9	0.62 ± 0.22
	180	90.4 ± 0.7	0.25 ± 0.02
SiMn	7	54.2 ± 0.6	–
	28	72.0 ± 0.3	1.70 ± 0.18
	90	76.9 ± 0.7	1.50 ± 0.52
	180	86.1 ± 1.2	0.74 ± 0.12

**Figure 2:**
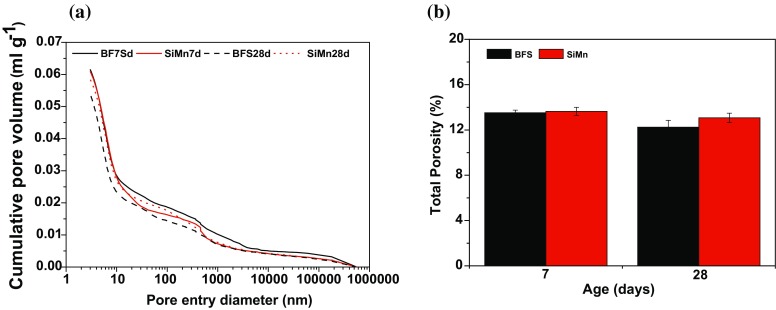
Mercury intrusion porosimetry results: **a** pore entry diameter distribution and **b** total porosity, for BFS and SiMn mortars

Table 2 also shows the chloride migration coefficient for alkali-activated BFS and SiMn mortars after 28, 90 and 180 days of curing. The inclusion of silicomanganese slag increased the *D*_nssm_ value by 17% in comparison with BFS mortars at 28 days, consistent with the porosimetric measurements in Fig. 2. There was in general a good agreement between the total porosity values and chloride migration coefficient values at this age. After 90 and 180 days, the BFS mortars also exhibited lower chloride migration coefficients than SiMn mortars. This parameter decreased with the curing time for both types of mortars, and there was a very low degree of chloride penetration into the samples at 180 days. The microstructure of the mortars changed over time, favoring the formation of denser C–(N)–A–S–H gels and restricting chloride penetration through the pore networks of the specimens. The *D*_nssm_ values for the blast furnace slag mortar are in agreement with the data observed by Ismail et al. [25].

The pore size distributions determined by MIP for BFS and SiMn mortars after 7 and 28 days of curing are depicted in Fig. 2a, and total porosity in Fig. 2b (average value of two measurements). The cumulative pore volume of BFS mortar at 7 days and SiMn mortar at both ages was larger than that of BFS mortar at 28 days. The improvement in the pore structure of the latter can be due to the higher degree of reaction, which produced additional Na-substituted calcium silicate hydrate rich in Al (C–(N)–A–S–H) gel, as the main reaction product, at late ages. The cumulative pore volume between 36 nm and 465 nm also decreased for this mortar; the activation of the slag was enhanced and the pore structure became more refined as these pores were replaced by pores with smaller entry diameters. The total porosity values for SiMn mortars were similar to those obtained for BFS mortars, independent of the time of curing (see Fig. 2b).

### Corrosion of steel rebars embedded in alkali-activated slag mortars

#### Evolution of corrosion potential

**Figure 3:**
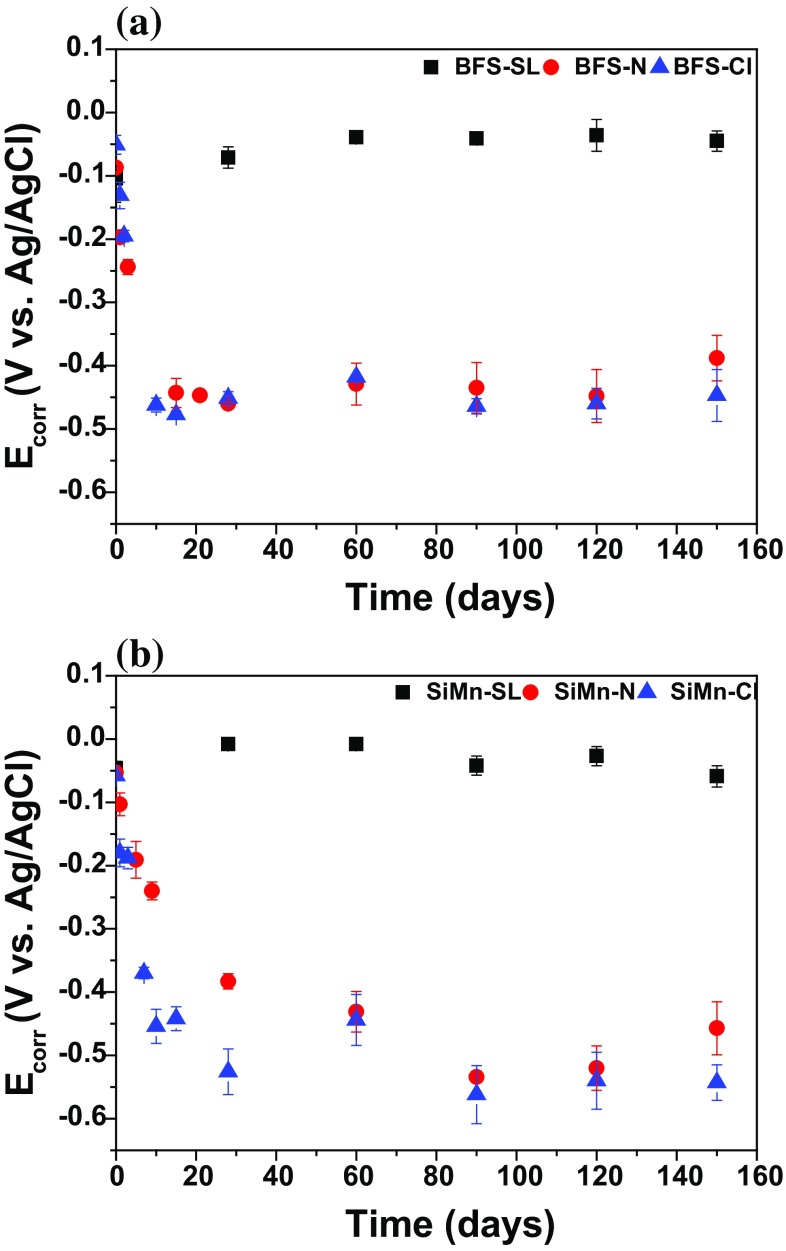
Evolution of the corrosion potential with time for 28-day cured alkali-activated BFS and SiMn mortars exposed to standard laboratory conditions (SL), alkaline solution (N) and chloride-rich solution (Cl) as a function of immersion time

The resistance to chloride-induced corrosion of alkali-activated BFS and SiMn mortars was assessed by monitoring *E*_corr_ for 150 days, after the samples had first been cured under sealed conditions for 28 days. The *E*_corr_ values obtained for both types of mortar are reported in Fig. 3. A set of specimens were immersed in alkaline solution (condition N) to give a reference baseline defining the behavior of steel in undamaged slag mortars. The rebars in these reference mortars presented *E*_corr_ values which decreased with time in both cases, but more rapidly for the BFS mortar (comparing filled and unfilled circles in Fig. 3): *E*_corr_ reached − 0.24 V at 3 days and − 0.45 V at 28 days (note that all *E*_corr_ values are given relative to Ag/AgCl), compared to − 0.24 V at 9 days and − 0.38 V at 28 days for SiMn. However, beyond this time, the mortars containing the SiMn slag continued to show a further decrease to − 0.54 V at 90 days. These very negative *E*_corr_ values would usually, in the analysis of such specimens, be taken to indicate the onset of corrosion attack on the steel, but no chloride was introduced into the specimens and the pH was maintained at approximately 14, so further analysis related to the redox chemistry of the binders themselves is required to determine the causes of these observations.

These results are likely to be related to the presence of sulfide in the slags used to produce the binders, and the slow oxygen reduction kinetics due to hindered mass transport. Much of the sulfur in the original slags was present as sulfide (− 2 oxidation state), which was released into the alkaline aqueous environment as the slag glasses dissolved during alkali activation. Hummel et al. [26] indicated by thermodynamic calculations that HS^−^ is the dominant sulfur species under highly alkaline conditions, which will generate reducing conditions. However, Gruskovnjak et al. [27] observed that SO_3_^2−^ may dominate the sulfur speciation under moderately reducing conditions due to kinetic effects, whereas under more strongly reducing conditions, S_2_O_3_^2−^, HS^−^ and a series of polysulfides will be dominant. The evolution of the speciation of sulfur during slag hydration has been determined by X-ray absorption near-edge structure (XANES) spectroscopy [13, 28], where it was observed that the sulfur in an anhydrous granulated blast furnace slag is mainly present in reduced form, S^2−^ and S^0^, while in the activation of the slag, those sulfur species react with oxygen and alkaline solutions to form S_2_O_3_^2−^ and SO_4_^2−^. The S_3_^−^ radical starts to form by reaction of sulfide (S^2−^) and elemental sulfur (S^0^) under an alkaline environment. The scavenging of oxygen by reduced sulfur compounds is undoubtedly important to the corrosion behavior identified here, but the sulfide itself will also cause changes in the nature and chemistry of the passive film on the steel, and this behavior is the focus of the current paper.

The specimens tested here were totally immersed in the alkaline-rich solutions throughout the specified durations, meaning that little or no oxygen would have entered into the specimens beyond that which was dissolved in the water, and thus, there was little scope for additional oxidation to be induced in this way. This led to strongly reducing conditions and therefore, the reduced forms of sulfur discussed above are expected to be the dominant species in these systems.

The presence of sulfide species can significantly reduce the redox potential of the pore solution of slag-rich cement mortars [29], making the redox potential of slag-containing cements around 400 mV lower than that of PC. Garcia et al. [30] recorded very low potentials (around − 490 mV vs. Ag/AgCl) after 1 or 2 days in blended concretes (40% PC + 60% blast furnace slag) due to intrinsic binder chemistry, not corrosion initiation. The drop in the redox potential is a result of the release of reductants from the slag during its reaction (whether induced by alkali activation or in PC blends), and the inability of the cathode to generate a current that overcomes the HS^−^ oxidation peak. Ma et al. [31] reported that the corrosion rate generally decreased with an increase in the concentration of sulfide due to the generation of a strongly reducing environment.

The rebars in the specimens immersed in the alkaline chloride-rich solution (denoted Cl; triangles in Fig. 3) presented *E*_corr_ values about − 0.131 V vs. Ag/AgCl for BFS mortar, and − 0.180 V for SiMn mortar, after 1 day of immersion. After 10 days, the *E*_corr_ values in both of these mortars continued to decrease and reached values of − 0.450 V or more active in the longer term (Fig. 3).

Corrosion initiation of steel rebar is generally considered to take place when the potential of the steel suffers a variation of about 250 mV [32], which is the difference usually encountered between steel in the passive state and in the active state. Nevertheless, for alkali-activated slag mortars, very low steel potential values were recorded before contamination by chlorides. These values remained below the expected pitting potential throughout the entire period of immersion, both with and without chloride, and therefore, it was difficult to conclude from these data whether corrosion was taking place, or whether the electrochemical results were instead dominated by the presence of sulfide species in the specimens. The values of corrosion potential for the SiMn-containing mortars were in general slightly lower in alkaline and chloride-rich solutions compared to those for BFS mortars, but all the *E*_corr_ values were in the region that is classified as very active [32].

The specimens exposed to standard laboratory conditions, independent of the slag used, showed much higher *E*_corr_ values (− 0.008 to − 0.104 V vs. Ag/AgCl), compared with specimens exposed to alkalis or chloride-rich solutions, which indicates that the steel was in a passive state [32]. These specimens were in open storage, in dry conditions and exposed to air, and so the oxygen diffused into the pore network and oxidized the sulfur species. Therefore, the depletion of reduced sulfur species meant that the electrochemical data were not influenced by their presence, and more positive *E*_corr_ values were obtained in these specimens. This was confirmed by visual observation of the color of the alkali-activated slag mortars, as outlined below.

#### Visual examination and corrosion product analysis

**Figure 4:**
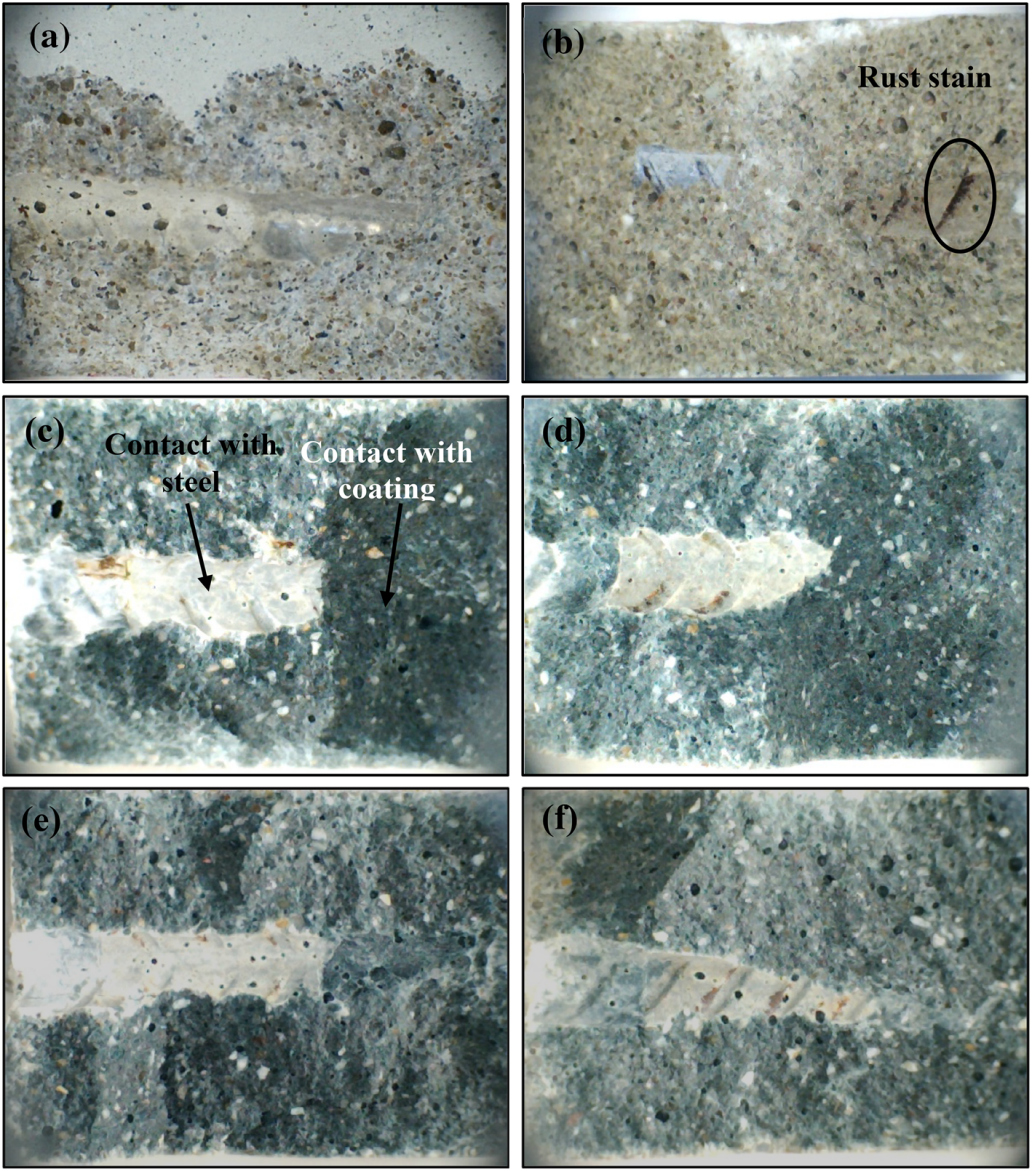
Mortar cover around the steel bars extracted from **a** BFS–SL, **b** SiMn–SL, **c** BFS–N, **d** SiMn–N, **e** BFS–Cl, and **f** SiMn–Cl mortars, after 150 days of exposure

The mortar cover immediately around the steel surface was evaluated after removal of the rebars after 150 days, Fig. 4. Similar results were obtained after 60 days, so the discussion here focuses on the 150-day samples. The BFS and SiMn mortars exposed to standard laboratory conditions were white, having lost their greenish color, Fig. 4a, b. The green color of the mortar was related to the presence of reduced sulfur species, particularly polysulfides [13, 28]. When the samples were exposed to air, the oxygen penetrated into the mortar, oxidized the sulfur species and destroyed the green coloration of the samples. This is often observed in slag-Portland cements as well as in AAS, but could not happen for immersed samples because they were not exposed to air. It is particularly striking that the green color in Fig. 4c–f is converted to be white only in the areas that were in direct contact with the steel. If sulfide was simply acting as an oxygen scavenger, the discoloration would be expected to take place throughout the bulk material as the only oxygen present was that which was dissolved in the pore solution. The loss of green coloration specifically at the steel–mortar interface (but not where the steel is coated with epoxy) indicates that the sulfide must have been consumed by reaction with the surface layer of the steel, and excludes the possibility that this is related in any way to a casting defect enabling oxygen ingress. Rust stains left by the original oxide on the steel rebar could be observed at the steel/mortar interface, as marked in Fig. 4b.

The BFS and SiMn mortars immersed in the alkaline and chloride-rich solutions maintained the green tonality. However, the color immediately at the steel/mortar interface was either white or a much lighter green. A proposed explanation for the loss of color could be a process of chemical reaction between dissolved (poly)sulfides and the rust layer. Where the rust layer (including Fe^3+^) and the pore solution of the mortar were in contact, the Fe^3+^ could act as an oxidant to consume the polysulfides from the solution, including some incorporation into an altered passive layer containing iron sulfide as well as the initial oxides and hydroxides. This consumption of the sulfide species (by oxidation and/or by incorporation) would then cause the visible discoloration around the steel.

Iron has a tendency to form FeS instead of iron oxide/hydroxide in the presence of S^2−^, according to the Pourbaix diagram for the Fe–S system [33, 34], and it is therefore highly likely that a complex iron sulfide layer exists on the surface at such negative potentials. The formation of this type of compound was also predicted through thermodynamic calculations in the hydration of alkali-activated slag by Lothenbach and Gruskovnjak [35]. Under reducing conditions, Fe^2+^ is favored over Fe^3+^, with Fe^3+^ prone to reduction by the partial oxidation of sulfide; the formation of mackinawite (FeS) was predicted in that study. Disordered mackinawite is expected to be the first iron sulfide formed at ambient temperature; it is stable for up to 4 months under reducing and alkaline conditions [36]. The formation of other FeS polymorphs such as pyrrhotite and troilite could also take place, while pyrite (FeS_2_) is not favored for kinetic reasons [37]. X-ray photoelectron spectroscopy indicated that the steel surface is rich in various Fe–S species when steel was immersed in simulated solutions of high-Ca alkali-activated materials [38], providing further support for this explanation of the observed behavior here.

**Figure 5:**
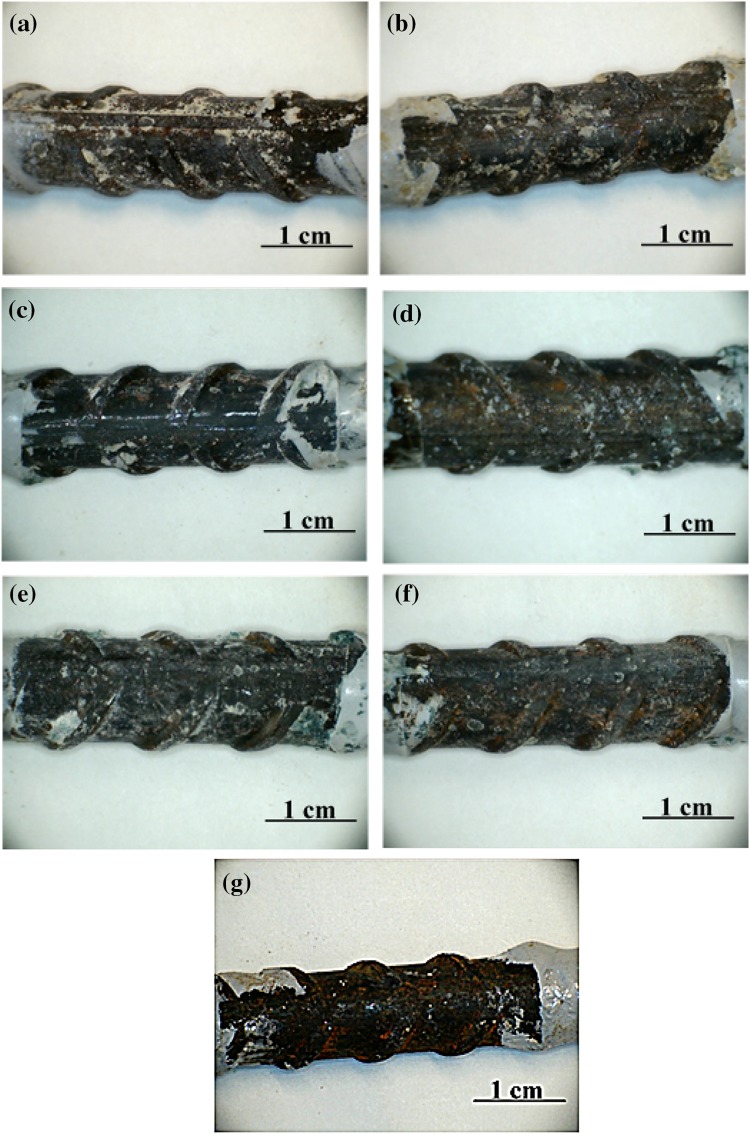
Photographs of steel bars extracted from the mortars after 150 days of exposure/immersion: **a** BFS–SL; **b** SiMn–SL; **c** BFS–N; **d** SiMn–N; **e** BFS–Cl; **f** SiMn–Cl and **g** the original rebar. The diameter of each bar is 12 mm

Figure 5 shows photographs of the steel specimens extracted from BFS and SiMn mortars exposed to different environments, to enable visualization of the progress of corrosion after 150 days. The surface aspect of the original rebar before embedding in the mortars is also shown. The steel bars extracted from the specimens had small fragments of mortars strongly adhering to their surfaces, and in some regions reddish stains, apparently distributed randomly, were also observed. These stains could correspond to the initial rust layer of the original rebar, Fig. 5g, or may be newly formed. The steel bars did not show visual evidence of pits or corrosion product layers, which was unexpected considering that they had been held at such low *E*_corr_ values for 150 days.

**Figure 6:**
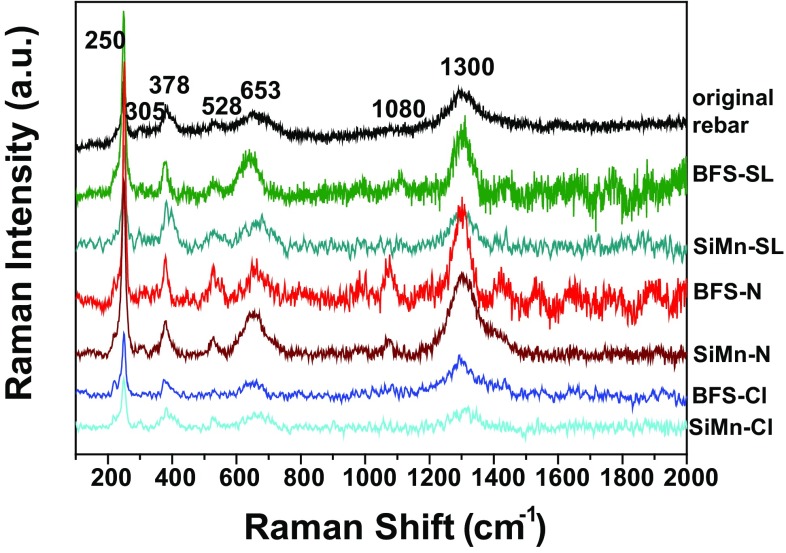
Raman spectra of the original steel surface, and the steel surface embedded in BFS and SiMn mortars exposed to standard laboratory conditions (SL), or immersed in alkaline (N) or chloride-rich solutions (Cl), for 150 days

To further investigate this, the nature of the corrosion products formed on the steel surface was studied through Raman spectroscopy. Figure 6 shows the Raman spectra of iron compounds formed on the rebars exposed at different environments after 150 days; similar spectra were obtained after 60 days. All spectra showed that the dominant iron compound formed on the steel surface was lepidocrocite (*γ*-FeOOH) on the original rebars, in the alkaline and alkaline chloride-rich solutions, and standard laboratory conditions. The well-defined peaks detected at 250 (most intense), 305, 378, 528, 653, 1080 and 1300 cm^−1^ indicate the presence of lepidocrocite [39, 40]. The observation of lepidocrocite as the only constituent phase of the rust layer formed on the steel exposed to the atmosphere is common, since it is generally agreed that crystalline lepidocrocite is the first phase to form on the steel surface [41].

The presence of different complex iron sulfides was difficult to confirm; for example, pyrrhotite Fe_7_S_8_ (RRuff Raman spectroscopy database ID #R060440) presents two Raman signals in its spectrum, at 345 and 378 cm^−1^ (the most intense) [42]. However, the overlap between these and the peaks of lepidocrocite meant that this characteristic peak was impossible to observe here. Moreover, mackinawite Fe_1+*x*_S (*x* = 0–0.07) (RRuff ID #R060388) usually presents a disordered structure which hinders its detection by Raman spectroscopy.

Under high alkalinity and chloride conditions, the steel embedded in BFS and SiMn mortars presented very negative *E*_corr_ values, indicating low resistance to corrosion, but the opposite was seen from the observation of the extracted rebars. Electrochemical impedance spectroscopy measurements and polarization curves were also carried out after 150 days of exposure, to try to explain why both results, open-circuit potential and visual examination, showed different behavior of the steel embedded in BFS and SiMn mortars in the presence of chloride ions.

#### Electrochemical impedance spectroscopy (EIS)

**Figure 7:**
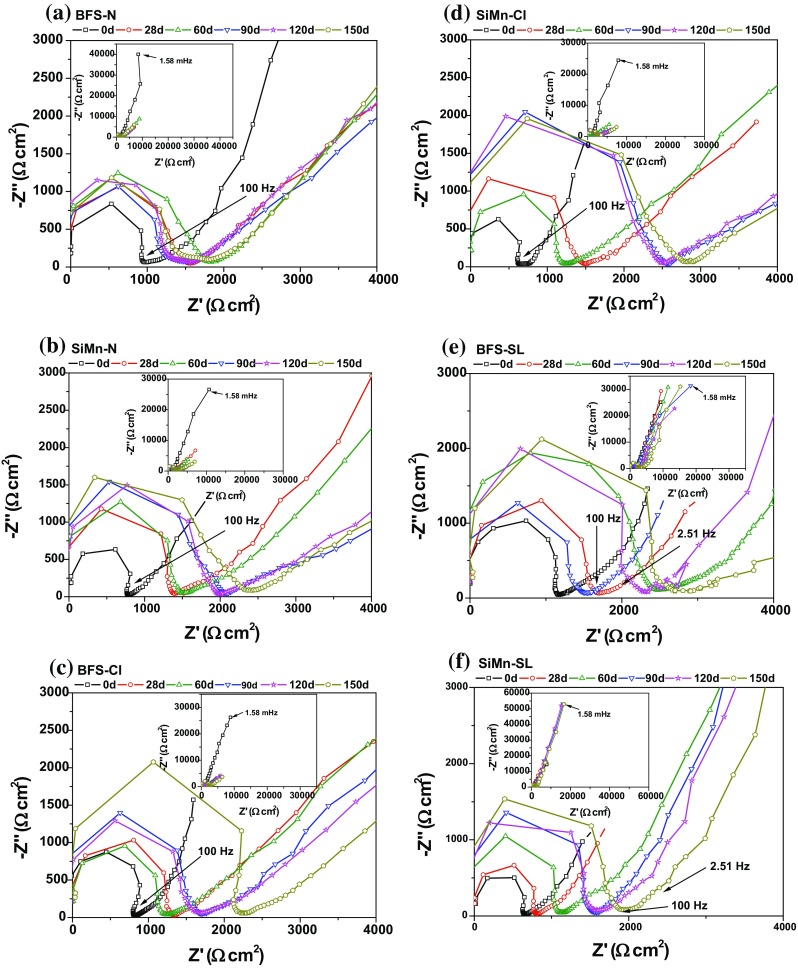
Nyquist plots for BFS and SiMn mortars after 150 days of immersion in **a** and **b** an alkaline solution (N) and **c** and **d** a chloride-rich solution (Cl) and **e** and **f** exposed to standard laboratory conditions (SL)

Nyquist plots recorded for BFS and SiMn mortars after 150 days exposed to different environments are shown in Fig. 7. The impedance spectra for the mortars immersed in alkaline (N) or chloride (Cl) solutions showed two capacitive arcs. A capacitive arc was observed at high frequency (usually in the 10^6^–100 Hz range), attributed to the dielectric properties of the bulk mortar [43, 44]. The impedance response at frequencies lower than 100 Hz was associated with the charge transfer reactions on the rebar surface, giving information on the corrosion process [43, 45, 46].

However, the Nyquist plot for the mortars exposed to standard laboratory conditions showed three capacitive arcs. The first time constant (at frequencies above 100 Hz) was associated with the dielectric properties of the bulk mortar, and the second time constant in the intermediate frequency range (usually in the 100–2.51 Hz range) was connected with the presence of the interface film [43, 45, 46]. The impedance response at frequencies lower than 2.51 Hz was associated with the steel surface corrosion processes.

**Figure 8:**
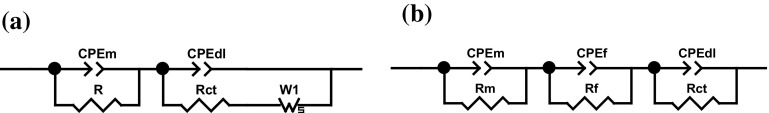
Equivalent electrical circuits (EECs) used in the fitting process for BFS and SiMn mortars: **a** immersed in the alkaline and alkaline-rich chloride solutions; and **b** exposed to standard laboratory conditions

These spectra were fitted by the electrical equivalent circuits (EEC) shown in Fig. 8, using the *Zview* software. The EEC in Fig. 8a is comprised of parallel resistance (*R*) and constant phase element (*CPE*) combinations, where two such combinations are arranged in series to construct the full EEC. The first combination (*R*-CPE_m_) is linked to the electrolyte (*R*_e_) and bulk matrix (*R*_m_) resistances and the bulk matrix capacitance [45, 46], respectively, where the total resistance *R* is given by 1/*R* = 1/*R*_e_ + 1/*R*_m_. The electrolyte and matrix resistances are assumed to act in parallel (and thus in parallel with the same CPE) because the specimens were totally immersed in the solution, and so the electrolyte was held within the bulk matrix. CPE_m_ corresponds to the dielectric properties of the mortar.

The second combination (*R*_*ct*_–*CPE*_*dl*_) was associated with the charge transfer resistance and double-layer capacitance of the surface of the rebars. This combination contained a finite-length Warburg (*W*_1_) element in series with the resistive element, representing the mass transport processes occurring at the mortar/metal interface.

In the EEC, the substitution of a pure capacitor by a constant phase element (CPE) was selected due to the inhomogeneities and discontinuities at interfaces [47]. The electrical impedance of a CPE is defined by *Z*_CPE_ = (*Y*)^−1^(*jω*)^−*α*^, where *Y* is the admittance, *ω* is the angular frequency (= 2π*f*, where *f* is the applied frequency), *j* is the imaginary unit (*j* ^2^ = − 1), and *α*, defined as the CPE exponent, is in the range − 1 ≤ *α* ≤ 1. When *α* = 0 the CPE is a resistor; when *α* = 1, it is a capacitor; when *α* = − 1, it is an inductor; and when *α* = 0.5, the CPE is a Warburg element. In this case, the relationship between *Y* and the Warburg coefficient (*σ*_W_) is given by $$ \sigma_{W} = {\raise0.7ex\hbox{${1^{{}} }$} \!\mathord{\left/ {\vphantom {{1^{{}} } {Y\sqrt 2 }}}\right.\kern-0pt} \!\lower0.7ex\hbox{${Y\sqrt 2 }$}} $$.

Conversely, the fitting of the impedance spectra for specimens exposed to standard laboratory conditions required the more complex EEC of Fig. 8b, with a series of three parallel resistance-constant phase element combinations. In this circuit, the first combination (*R*_m_–CPE_m_) is related to the bulk matrix resistance and capacitance, while the second combination (*R*_f_–CPE_f_) is associated with the mortar/steel interface film resistance and capacitance, and finally, the (*R*_ct_–CPE_dl_) combination is related to the charge transfer resistance and double-layer capacitance, but not including a finite-length Warburg element. In this system, the mortars are in direct contact with air, the oxygen reacts with the sulfides, and there is no diffusion of these species in the medium. Therefore, it was not necessary to include a diffusion element in the equivalent electric circuit, but a new combination of a resistance and a constant phase element must be introduced to obtain adequate fitting of the impedance spectra. The same circuit was used to study the electrochemical characteristics of reinforced mortar corrosion in the presence of chloride ions [48]

**Figure 9:**
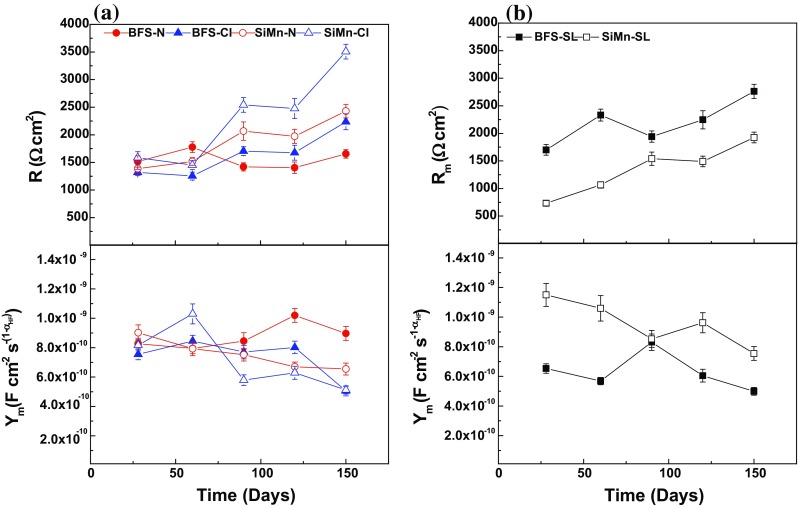
Comparison of the bulk matrix electrical parameters of BFS and SiMn mortars **a** immersed in alkaline and chloride-rich solutions and **b** exposed to standard laboratory conditions

Figure 9 displays the changes in the electrochemical parameters of the first combination (*R*–CPE_m_) for conditions N and Cl, *R*_m_–CPE_m_ for condition SL), describing the response at high frequencies, as a function of time. For both immersed mortars, *R* increased and CPE_m_ (*Y*_m_) decreased, respectively, with the time of immersion. These trends were not so clear for SiMn mortar immersed in the alkaline solution, where both parameters remained practically constant. Such evolution over time is attributed to the ongoing mortar curing, inducing densification of the specimens and therefore, a higher resistance to the penetration of electrolytes containing aggressive ions. This higher resistance over time is expected if the porosimetry and chloride migration coefficient data are considered. The total porosity decreased with the curing time (Fig. 2) in the same way as *D*_nssm_ (Table 2) as reaction progresses.

The mortars exposed to alkaline solution presented lower *R* values and higher CPE_m_ (*Y*_m_), probably connected with the easier penetration of hydroxyl ions than chloride ions through the bulk mortar; the ionic mobility of OH^−^ is 20.64 × 10^−8^ m^2^ s^−1^ V^−1^ in water at 25 °C, while that of Cl^−^ is lower, 7.92 × 10^−8^ m^2^ s^−1^ V^−1^ [49]. Regarding the type of mortar, the mortar containing 10% SiMn slag presented higher *R* values after immersion in either solution. The effect of the composition was not so clear in the CPE_m_ (*Y*_m_) parameter, although, in general terms, the presence of silicomanganese slag gave lower values after 90 days. Although the cumulative pore volume and total porosity values were slightly higher for SiMn mortars than BFS mortars, these differences are not very significant between both mortars, and the SiMn mortars presented a higher resistivity. A possible explanation could be related to differences in the chloride binding capacities of each of the types of mortars studied here; if this was higher in SiMn mortars, this would be expected to lead to higher R values, but no data are yet available to enable this theory to be confirmed or disproven.

Figure 9b shows the evolution of the bulk matrix resistance (*R*_m_) and capacitance (*Y*_m_) over time, for both mortars exposed to standard laboratory conditions. An increase in the exposure time led to an increase in the *R*_m_ parameter and a decrease in the *Y*_m_ parameter due to the loss of solution from inside the pores of the mortars with time, meaning that the movement of ionic species was restricted.

**Figure 10:**
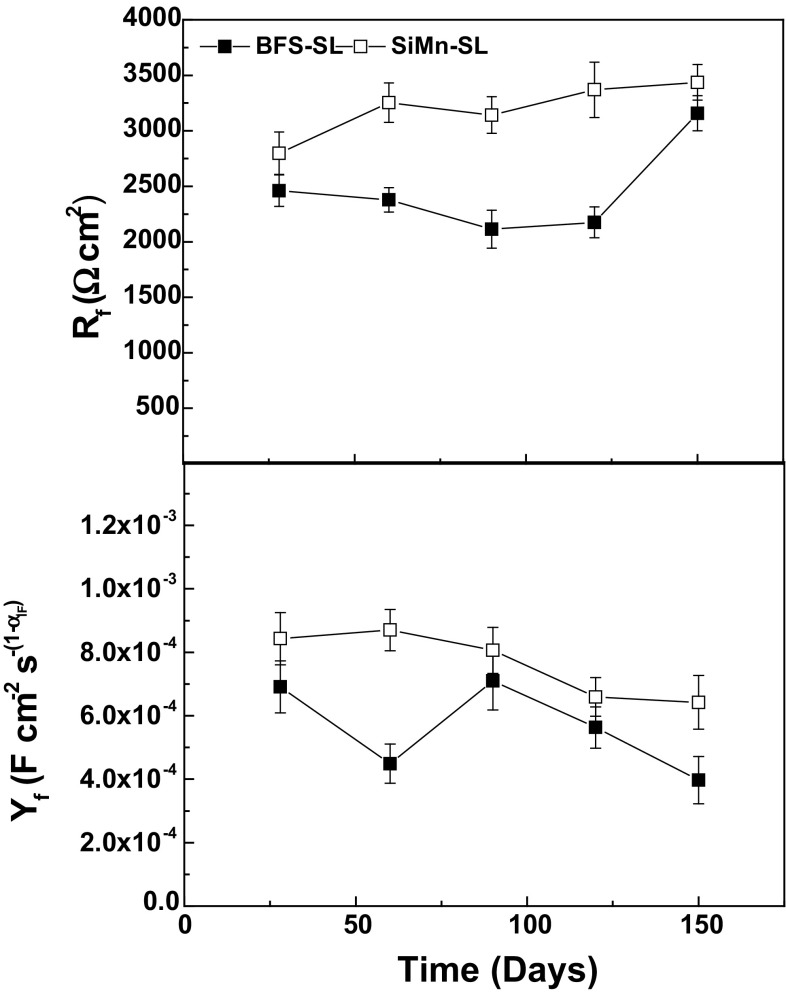
Comparison of *R*_f_ and *Y*_f_ parameters of steel embedded in BFS and SiMn mortars exposure to standard laboratory conditions

Figure 10 displays the changes in the steel/concrete interface film resistance (*R*_f_) and capacitance CPE_f_ (*Y*_f_) for BFS and SiMn mortars exposed to standard laboratory conditions. The formation of the film was favored by longer exposure times as the alkaline binder and the steel continued to interact. So, an increase in its thickness took place, making both mortars exhibited higher values of *R*_f_ and lower values of CPE_f_ (*Y*_f_) at 150 days.

The film formed on the steel surface of SiMn mortars was more protective and more compact, with *R*_f_ values of 3500 Ω cm^2^ being much higher than those obtained for BFS mortars. This behavior could be related with a more saturated pore network over time, leading to the formation of a thick passive film. *Y*_f_ values were in the range of 397–870 µF cm^−2^ s^−(1−αIF)^, in reasonable agreement with other studies in the literature [50, 51].

**Figure 11:**
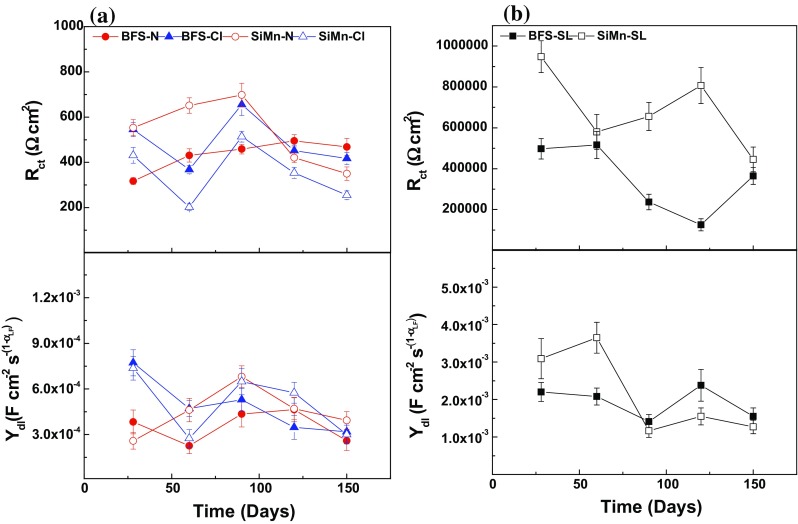
Comparison of *R*_ct_ and *Y*_dl_ parameters of steel embedded in BFS and SiMn mortars immersed in alkaline and chloride-rich solutions (**a**) and exposed to standard laboratory conditions (**b**)

Figure 11 shows the evolution of the charge transfer resistance (*R*_ct_) and double-layer capacitance CPE_dl_ (*Y*_dl_) of the surface rebars as a function of time for mortars immersed in alkaline and chloride-rich solutions (Fig. 11a), and exposed to standard laboratory conditions (Fig. 11b). In immersed mortars, *R*_ct_ values were between 200 and 700 Ω cm^2^. There was not a clear trend in these values either between samples or as a function of time, but they were small, meaning that the charge transfer did not control the corrosion rates. This was also evidenced by the constancy of the double-layer capacitance parameter, which remained in the range from 2.3 to 7.7 × 10^−4^ F cm^−2^ s^−(1−αdl)^, throughout immersion. The presence of chlorides and the composition of the mortar did not seem to affect the evolution of *R*_ct_ and *Y*_dl_ parameters in these conditions.

For the mortars exposed to standard laboratory conditions, *R*_ct_ values were higher than those obtained for the mortars immersed in alkaline solutions with or without chloride (see Fig. 11a). These values remained between 200 and 900 kΩ cm^2^ until the end of the test, indicating that the charge transfer through the passive film controlled the corrosion process. SiMn mortars exhibited higher *R*_ct_ values, related to an increase in the protective nature of the surface film. The capacitance associated with the CPE_dl_ varied between 1.2 and 3.6 × 10^−3^ F cm^−2^ s^−(1−αdl)^, demonstrating minimal changes in the double layer at the passive film/steel interface for BFS and SiMn mortars.

**Figure 12:**
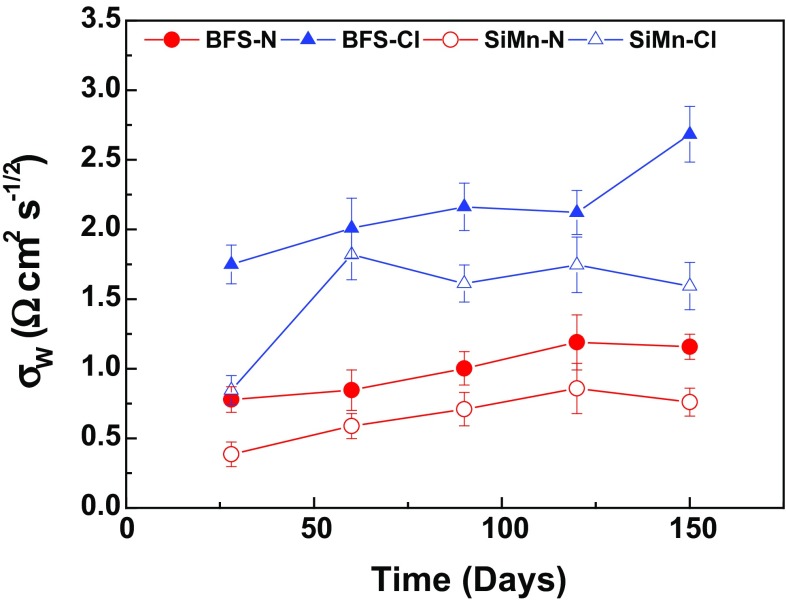
Evolution of the σ_w_ parameter for BFS and SiMn mortars immersed in both solutions with the time of immersion

Figure 12 shows the evolution of the Warburg diffusion element (*σ*_w_) for BFS and SiMn mortars during immersion in both solutions. A rise in the Warburg element, representing resistance to diffusion processes at the mortar/metal interface, was observed after 150 days of exposure. Mass transport through the mortar to the metal was impeded with increasing exposure time; this is agreement to the trend in chloride migration coefficients of the mortars, Table 2, which could be attributed to a refinement of the pore network with increasing age of the mortar. In the chloride-rich solution, the chloride could break down the passive film of the steel, leading to easier transport of ions and a decrease in the *σ*_w_ parameter. However, the mortars immersed in alkaline solution did not undergo diffusion of chloride, but a transport of ions did take place. These ions were the sulfide and polysulfides, present in the composition of the slags, which diffused to the surface of the steel and could react with the iron species. In this study, the *σ*_w_ values were higher in the chloride-rich solution, indicating the transport rates were lower. This could indicate that the corrosion process did not take place and the penetration of chloride across the passive film did not occur, in agreement with the photographs of steel bars extracted for both mortars after 150 days of immersion, Fig. 5. If the chloride migration coefficients are considered, where SiMn mortars show a higher degree of chloride penetration through the pore network of the specimens, they agree with the electrochemical results obtained.

#### Polarization curves

**Figure 13:**
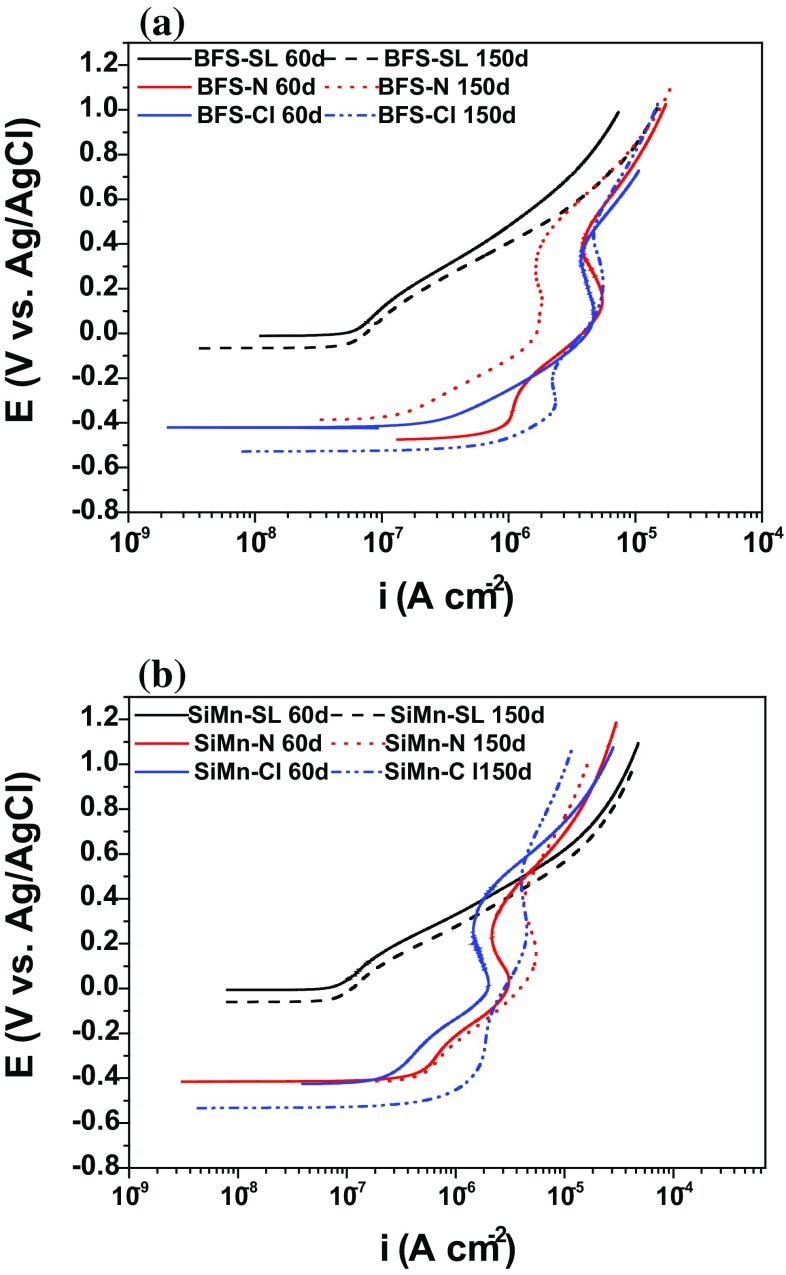
Anodic polarization curves recorded for steel embedded in **a** BFS and **b** SiMn mortars, after 60 and 150 days under standard laboratory conditions (SL), immersion in an alkaline solution (N) and in a chloride-rich solution (Cl)

Anodic polarization curves recorded for the steel rebars embedded in BFS and SiMn mortars exposed to the three different environments after 60 days and 150 days are shown in Fig. 13. The criteria used to analyze these results are conventionally based on the state of corrosion of steel in Portland cement-based concretes reported in [52], where an *i*_corr_ of < 0.1 µA cm^−2^ corresponds to passivity, 0.1 µA cm^−2^ < *i*_corr_ < 0.5 µA cm^−2^ corresponds to low corrosion, 0.5 µA cm^−2^ < *i*_corr_ < 1.0 µA cm^−2^ to high corrosion, and *i*_corr_ > 1.0 µA cm^−2^ to very high corrosion.

The curves in Fig. 13a for BFS mortars exposed to standard laboratory conditions indicated the presence of stable passive films, inducing noble *E*_corr_ (around − 0.01 to − 0.066 V vs. Ag/AgCl) and low *i*_corr_ values (~ 0.06 µA cm^−2^).

Conversely, in the alkaline solution (N), carbon steel embedded in BFS mortars (Fig. 13a) presented *E*_corr_ around − 0.475 V vs. Ag/AgCl and corrosion current density (*i*_corr_) ≈ 0.7 µA cm^−2^ after 60 days of immersion, which would usually indicate corrosion rather than passive behavior according to these criteria. However, this is not straightforwardly reconciled with the fact that the samples were not exposed to aggressive species that could damage the passive layer. An active/passive transition occurred in the anodic branch, with the passive potential in the range − 0.308 to − 0.173 V vs. Ag/AgCl, which could be associated with the transformation of S^2−^. The sudden increase in the current density for all scans shown in Fig. 13a at a potential of about + 0.55 V vs. Ag/AgCl is attributed to oxygen evolution at this high imposed potential [53].

However, after 150 days of immersion, *E*_corr_ was ennobled up to − 0.386 V vs. Ag/AgCl and the *i*_corr_ value was around 0.3 µA cm^−2^, closer to the values that would be expected for steel in a passive state [52]. This behavior could be associated with the consumption of sulfide by formation of an AF_m_ type phase (e.g., C_3_A·2CaS·10H_2_O) [30, 54, 55], as the activation reaction continued taking place with the time; these phases can show an intense green color. The electrochemical data are strongly influenced by the presence of sulfide.

At later ages of the evolution of slag-containing cements, oxidation from HS^−^ to higher sulfur oxidation states, up to and including sulfate, and the uptake of these species by solids, are very important [13, 54, 56]. Due to these simultaneous processes of release and consumption, the HS^-^ concentration is not directly correlated with the degree of slag dissolution in a more mature binder system. Typical anions frequently found in AF_m_ phases are OH^−^, SO_4_^2−^ and CO_3_^2−^, and under reducing conditions, HS^−^ or S_2_O_3_^2−^ can be incorporated or ion-exchanged onto the anion sites [13].

Figure 13a also shows the polarization curves measured for the samples immersed in the alkaline chloride solution for 60 days, which showed *E*_corr_ around − 0.423 V versus Ag/AgCl and *i*_corr_ ~ 0.9–1.0 µA cm^−2^. These corrosion current densities appear to indicate a high corrosion rate of the steel, but this stands in contrast to the lack of corrosion observed in the photographs of the steel in Fig. 5. After 150 days, the anodic polarization curves for these specimens exhibited much more active *E*_corr_ (− 0.528 V vs. Ag/AgCl) and much higher *i*_corr_ values (1.2 µA cm^−2^), suggesting that a degradation of surface film protectiveness was taking place, as chloride accumulated over time. It has been observed [57] in simulated alkali-activated slag pore solutions, that both the concentration of sulfide in the pore solution and the time of exposure played a critical role in defining the onset of chloride-induced pitting on the steel surface, due to the alteration of the passive film in the presence of a strong reductant such as sulfide. Here, current is flowing in the mortar, but the corrosion process evidently does not take place, and this will be discussed in more detail in the “Implications for the stability of steel in alkali-activated slag mortars” section. An active/passive transition with passive potentials in the range − 0.316 to − 0.157 V vs. Ag/AgCl was observed.

The anodic polarization curves recorded for the SiMn-containing mortar immersed in an alkaline solution exhibited a shift of *E*_corr_ values from −0.455 to −0.413 V vs. Ag/AgCl when the samples were immersed for 150 compared to 60 days, while *i*_corr_ was unchanged at 0.4 µA cm^−2^. This value of *i*_corr_ was slightly lower than that obtained for BFS mortars after 150 days.

Figure 13b also shows the curves recorded for SiMn samples in chloride solutions, which provide evidence that at increasing exposure times, there was more extensive degradation of the surface passive film by the aggressive species, inducing more negative *E*_corr_ (around − 0.532 V vs. Ag/AgCl) and high *i*_corr_ values (1 µA cm^−2^), and consistent with Fig. 5f. After 150 days, an active/passive transition with passive potentials in the range − 0.320 to − 0.009 V vs. Ag/AgCl was observed. The shape of the curve was not notably different from that of BFS mortars at the same age. The presence of a larger amount of manganese in the mortar did not seem to have a meaningful effect on the potential and current density values.

### Implications for the stability of steel in alkali-activated slag mortars

The comparison between electrochemical results and characterization of extracted steel specimens for alkali-activated mortars, as outlined above, provides some apparent contradictions and the need for detailed further analysis. The electrochemical results indicated that corrosion attack was taking place to a moderate to high degree in the steel embedded in BFS and SiMn mortars immersed in either alkaline or alkali-chloride solutions, with *E*_corr_ values, and high *i*_corr_ values. However, the visual observation of the steel and the mortar at the end of the exposure period, and the analysis of corrosion products present on the steel surface through Raman spectroscopy, showed the opposite. The only iron species detected was the initial rust layer on the steel, which appeared to have been undamaged and was spectroscopically similar to the as-received steel surface. The apparent discrepancies between these results could be due to the presence of reduced sulfur in the slags. As discussed previously, the presence of sulfide anions can significantly reduce the redox potential of the pore solution around the rebar, which could react with the iron species to form iron sulfides, further passivating the surface steel and hindering the corrosion process. If the high observed corrosion currents are related to aqueous-phase rather than steel oxidation processes (i.e., the chemical reactions in the pore solution involving sulfide species), and this is the reason for the apparent mismatch between the electrochemical measurements and the actual observations of minimal corrosion, this will have important implications for the electrochemical assessment of corrosion in slag-containing cements as it means that the commonly applied criteria for determination of corrosion probability are not applicable for mortars and concretes made using these cements.

## Conclusions

Alkali-activated blast furnace slag mortars containing 10% silicomanganese slag presented lower compressive strength, higher cumulative pore volume and higher chloride migration coefficient values than those produced using BFS as the sole precursor in alkali activation. The silicomanganese slag is less reactive and has a larger particle size than the blast furnace slag used.

Under highly alkaline conditions, BFS and SiMn mortars presented very negative *E*_corr_ and high *i*_corr_ values, indicating that the sulfide supplied by the slags reduced the redox potential of the pore solution of both types of alkali-activated slag mortars.

When immersed in alkaline, chloride-rich solutions, both mortars exhibited similar values of these two electrochemical parameters in comparison with those obtained in alkaline solutions without chloride. This would usually be taken to indicate a lower resistance to corrosion processes, induced by the presence of chlorides, but the steel bars extracted from BFS and SiMn mortars did not show evident pits or corrosion product layers, indicating that the presence of sulfide reduces the redox potential in a way which offers protection from chloride-induced corrosion. This highlights the finding that it is necessary to reconsider some of the established criteria used to assess the likelihood of corrosion when studying cements that are rich in redox-active elements (in particular the sulfide supplied by blast furnace slag), as significant “corrosion current” (*i*_corr_) values can be observed due to the redox chemistry of aqueous sulfur, without any significant corrosion processes taking place on the surface of the steel itself. The presence of a high amount of MnO in the slag did not significantly affect the corrosion process of the steel under the conditions tested, although some diffusional processes were influenced by the differences in pore structure between the samples. There may be a critical sulfide concentration controlling corrosion kinetics, which does not allow the observation of the influence (if any) of Mn on redox processes.
